# Esophageal cancer with severe funnel chest treated by simultaneous funnel chest surgery and thoracoscopic esophagectomy: a case report

**DOI:** 10.1186/s12885-018-5145-4

**Published:** 2018-12-04

**Authors:** Keiichiro Hatoyama, Yusuke Taniyama, Tadashi Sakurai, Chiaki Sato, Hiroshi Okamoto, Yu Onodera, Takashi Kamei

**Affiliations:** Department of Surgery, Tohoku University Graduate School of Medicine, 1-1, Seiryo-machi, Aoba-ku, Sendai, Japan

**Keywords:** Esophageal cancer, Funnel chest, Thoracoscopic esophagectomy

## Abstract

**Background:**

Funnel chest is the most common chest deformity, occurring in 0.06–0.3% of the general population. When it occurs concomitantly with esophageal cancer, it hinders intrathoracic surgery that is necessary for treatment. Although there are a few reports of esophagectomy performed in patients with funnel chest, simultaneous treatment of esophageal cancer and funnel chest with funnel chest surgery (Nuss method) and esophagectomy has not been reported. We report the first case of advanced esophageal cancer complicated by severe funnel chest that was treated using the Nuss method and radical thoracoscopic esophagectomy.

**Case presentation:**

A 59-year-old man was diagnosed with advanced thoracic esophageal cancer and severe funnel chest. Because his sternum was almost attached to the vertebral bone, thereby creating a narrow space in the mediastinum, esophageal surgery was expected to be complicated. After the patient underwent neoadjuvant chemotherapy, we used the Nuss method to reconstruct the chest and widen the mediastinum. Subsequently, radical thoracoscopic esophagectomy was performed with the patient in the left decubitus position without any difficulty, and the postoperative course was uneventful.

**Conclusion:**

Simultaneous funnel chest surgery (Nuss method) and thoracoscopic esophagectomy with the patient in the left decubitus position are recommended for esophageal cancer patients with severe funnel chest.

## Background

Funnel chest is the most common chest deformity, occurring in 0.06–0.3% of the general population [1]. Although patients with funnel chest are generally asymptomatic, some with severe deformity experience chest pain and cardiopulmonary dysfunction [1]. In recent decades, surgical treatment for funnel chest has changed. Treatment previously included sternal turnover, which involves cutting the ribs and turning the sternum, and the Ravitch procedure, which involves deformation of the cartilage. However, these two procedures are relatively invasive due to the long incisions created on the anterior chest. The Nuss method, reported by Nuss et al. [2] in 1998, involves reconstructing the anterior chest wall with a metal bar in the anterior mediastinum and flipping the sternum upward. This bar must remain inside the patient for a few years to help reconstruct the shape of the sternum. Although the bar must be removed later, the procedure reduces massive surgical stress. When esophageal cancer occurs with funnel chest, the narrow working space in the mediastinum caused by funnel chest makes it difficult to perform intrathoracic surgery, which is necessary for treatment. We report the first case of advanced esophageal cancer complicated by severe funnel chest that was treated with the Nuss method and radical thoracoscopic esophagectomy [3].

### Case presentation

**Fig. 1:**
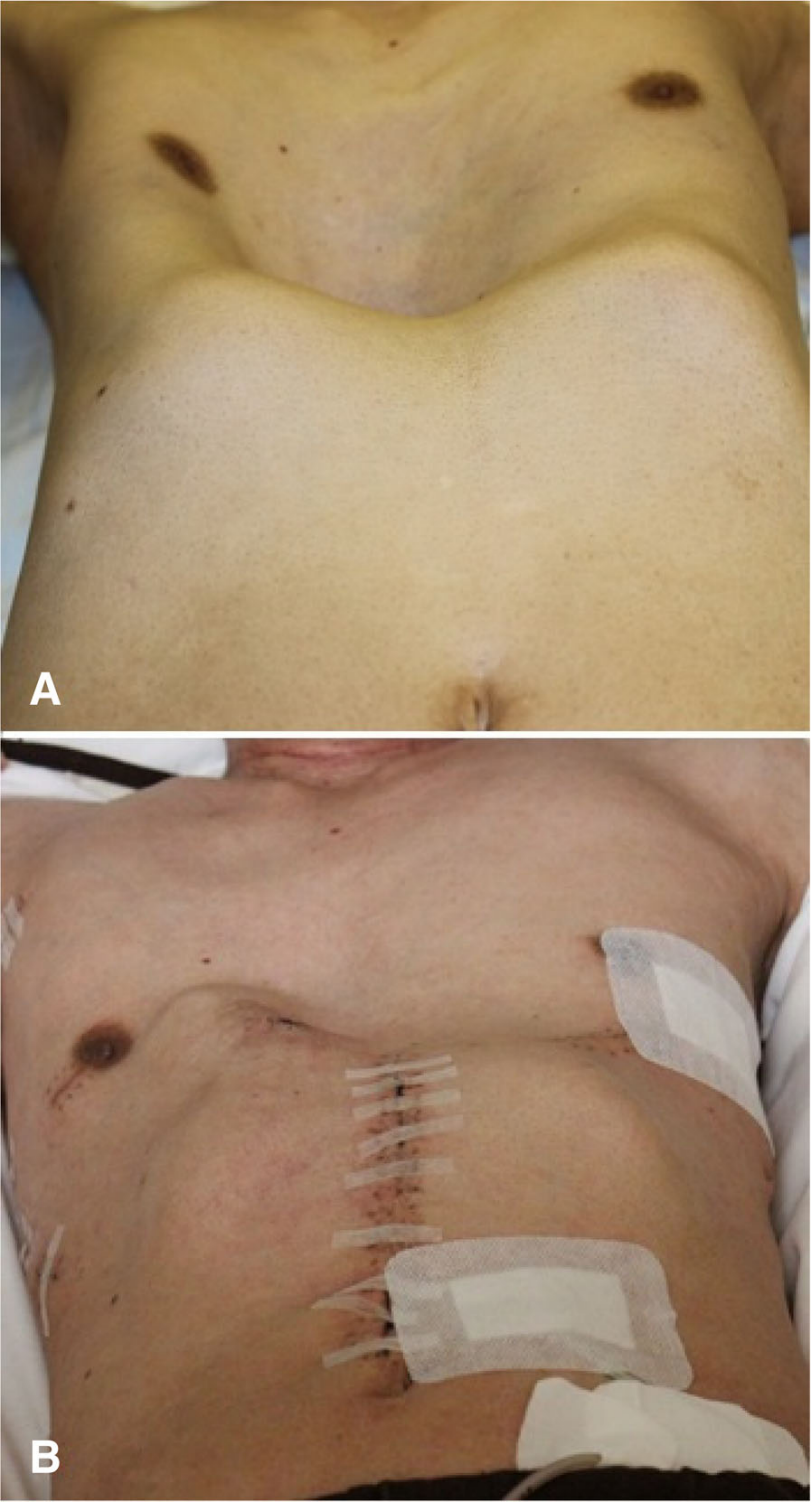
Physical examination of the patient **a** Before surgery. Funnel chest was apparent on physical examination.**b** After surgery.

**Fig. 2:**
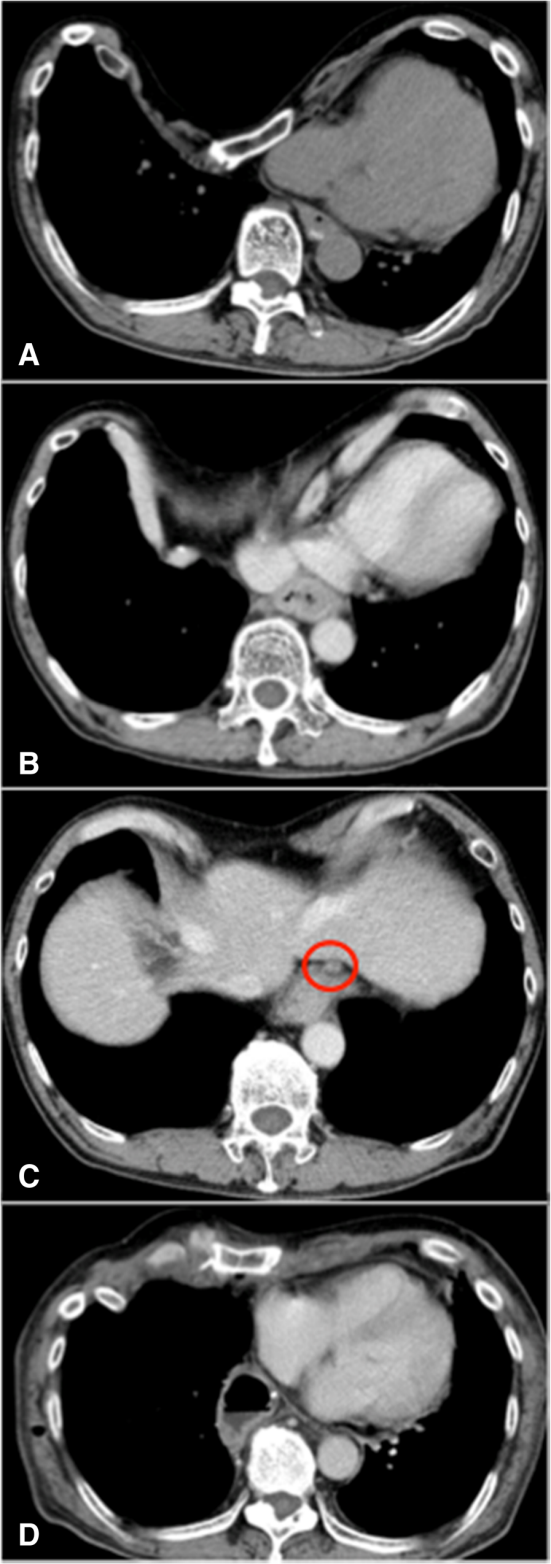
Chest computed tomography scan **a** Funnel chest (Haller index, 9.9).**b** Thickening of the esophagus.**c** The swollen lymph node (red circle).**d** After funnel chest surgery (Nuss method).

A 59-year-old man presented to the clinic with difficulty swallowing. An endoscopic study revealed an ulcerative esophageal tumor 36–40 cm from the upper incisors, and biopsy results indicated squamous cell carcinoma. He also had severe funnel chest, and his sternum was almost attached to the vertebral bone (Fig. 1a). He previously noticed this chest deformity but had no symptoms such as chest pain; therefore, he had not undergone medical examination for this condition. The Haller index (i.e., the distance of the inner rib cage divided by the distance between the sternal notch and the vertebrae [4]) was 9.9 (Fig. 2a). According to the UICC-TNM classification (version 7), the final preoperative diagnosis was stage IIIA, squamous cell carcinoma (cT3, cN1, cM0) (Fig. 2b, c). Although he had severe funnel chest, the preoperative examination revealed that his condition was generally good and he was fit to undergo surgery under general anesthesia. Two courses of 5-FU (800 mg/m2)/cisplatin (80 mg/m2) were administered as standard neoadjuvant chemotherapy. Then, we planned to perform funnel chest surgery (Nuss method) before esophagectomy, in order to achieve a suitable width in the mediastinum to allow for thoracoscopic surgery.

First, two convex metal bars were inserted under the sternum through small bilateral thoracic incisions. The bars were inserted with the convexity facing posteriorly. When the bars were in position, they were turned over to reconstruct the sternum and widen the mediastinum so that esophagectomy could be performed (Fig. 1b). Radical thoracoscopic esophagectomy with three-field lymph node dissection was performed with the patient in the left decubitus position, followed by gastric conduit reconstruction through the posterior mediastinum route. Surgery was performed without any complications, and the postoperative course was uneventful. Pathological staging according to the UICC-TNM classification (version 7) indicated stage IIIA (pT3, pN1, cM0). The metal bars were removed 1 year after surgery. The patient was in good condition at the 2-year follow-up examination.

### Discussion and conclusion

**Fig. 3:**
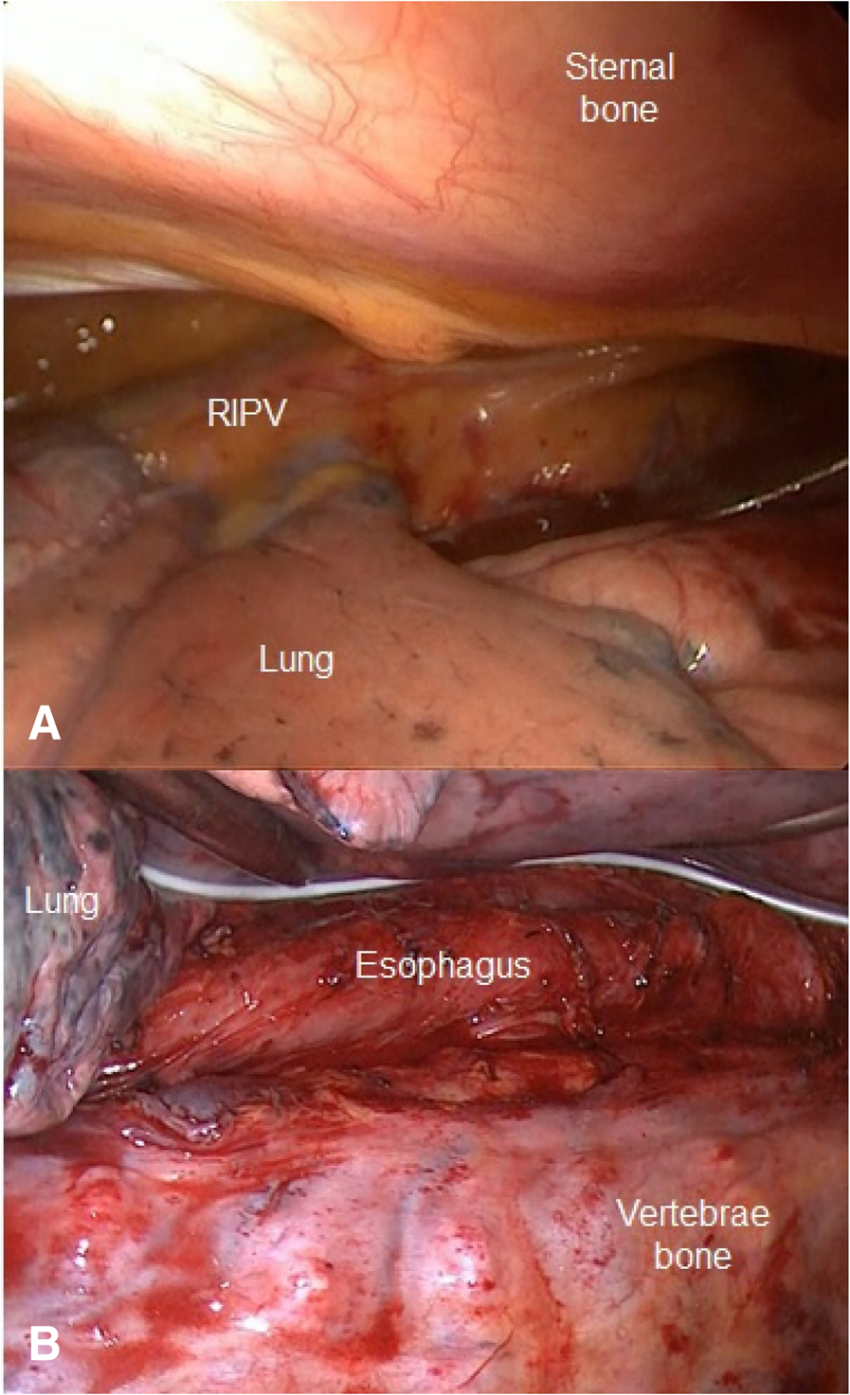
Intraoperative thoracoscopic findings **a** View of the anterior mediastinum before the funnel chest surgery, showing the narrow space of the mediastinum. RIPV: right inferior pulmonary vein. **b** Intraoperative findings of the radical thoracoscopic esophagectomy.

The funnel chest deformity creates a narrow operative space in the mediastinum. When funnel chest occurs concomitantly with esophageal cancer, this narrow space creates difficulty performing intrathoracic surgery, which is necessary for treatment. To the best of our knowledge, there are only a few reports of patients with funnel chest who have undergone esophagectomy. Iwata et al. [5] reported a case of esophageal cancer with funnel chest (Haller index, 4.6) that was treated by radical esophagectomy and right thoracotomy after the Ravitch procedure was performed to treat funnel chest. Takemura et al. [6] reported a similar case (unknown Haller index) that was treated with video-assisted thoracoscopic esophagectomy after sternal turnover with the patient in the right decubitus position, to treat funnel chest. This was performed because reconstruction using a gastric conduit through the posterior sternum route was not possible without funnel chest repair. These two procedures are relatively invasive due to the long incisions created on the anterior chest. Sato et al. [7] reported a similar case (Haller index, 4.83) that was treated with video-assisted thoracoscopic esophagectomy without funnel chest repair. In our case, however, it was impossible to perform thoracoscopic surgery without funnel chest repair because the chest deformity was so severe; his sternum was almost attached to the vertebral bone, making it difficult to visualize the surgical field, especially the middle to lower mediastinum (Fig. 3a). Therefore, funnel chest surgery (Nuss method) and thoracoscopic esophagectomy were performed simultaneously. Because the Nuss method allowed the patient to be in the decubitus position after the procedure, the view of the mediastinum was suitable and we were able to perform thoracoscopic esophagectomy (Figs. 2d and 3b). Interestingly, the normal chest and funnel chest do not appear to have any differences in terms of visibility and operability in the upper mediastinum [7].

Recently, thoracoscopic esophagectomy has been performed with the patient in the prone position at many centers because of its low incidence of respiratory complications [8]. However, the prone position is not appropriate immediately after the Nuss procedure because the metal bars would slide out due to chest wall compression. Additionally, right thoracotomy is not recommended because the incision line would be across the metal bars; without the surrounding tissue, the sternum would flip back to the dorsal side.

Anterior thoracic, posterior sternum, and posterior mediastinum routes are used for reconstruction during surgical treatment of esophageal cancer. The posterior sternum route is often selected because it allows easier access in the case of leakage compared with the posterior mediastinum route, and it has better cosmesis compared with the anterior thoracic route. However, this reconstruction route is inadequate for patients who have undergone the Nuss method because the metal bars settle behind the sternum. Contact and removal of the metal bars would increase the risk of damage to the reconstruction conduit. Therefore, the posterior mediastinum route was selected in our case.

Although possible infection caused by the metal bar was a concern, it was not appropriate to perform only funnel chest surgery and postpone esophagectomy because of the risk of esophageal cancer progression. Furthermore, precise evaluation of esophageal cancer using computed tomography would have been difficult after the metal bar had settled in the anterior chest. Therefore, we planned to perform simultaneous funnel chest surgery and radical esophagectomy. To avoid infection due to anastomosis leakage, McKeown esophagectomy was selected for reconstruction. Moreover, the metal bar was placed at the anterior chest, which was far from the anastomosis site. Although the possibility of infection remained, we assumed that this possibility was not high.

We recommend simultaneous funnel chest surgery (Nuss method) and thoracoscopic esophagectomy with the patient in the left decubitus position along with reconstruction using the posterior mediastinum route for esophageal cancer patients with severe funnel chest.
